# Supplementary material for: Splice-Junction-Based Mapping of Alternative Isoforms in the Human Proteome
Source: Cell Rep. Author manuscript; Available in PMC 2020 Jan 15. (PMC6961840; doi:10.1016/j.celrep.2019.11.026)
Supplement: 2 [file NIHMS1546469-supplement-2.zip › DF1/PXD006675/RightAtrium/RightAtrium_33_CCS_GSGQLRVQDVEVHLEDQMVLVHTTLPSQEVQALLEGTGR.pdf]

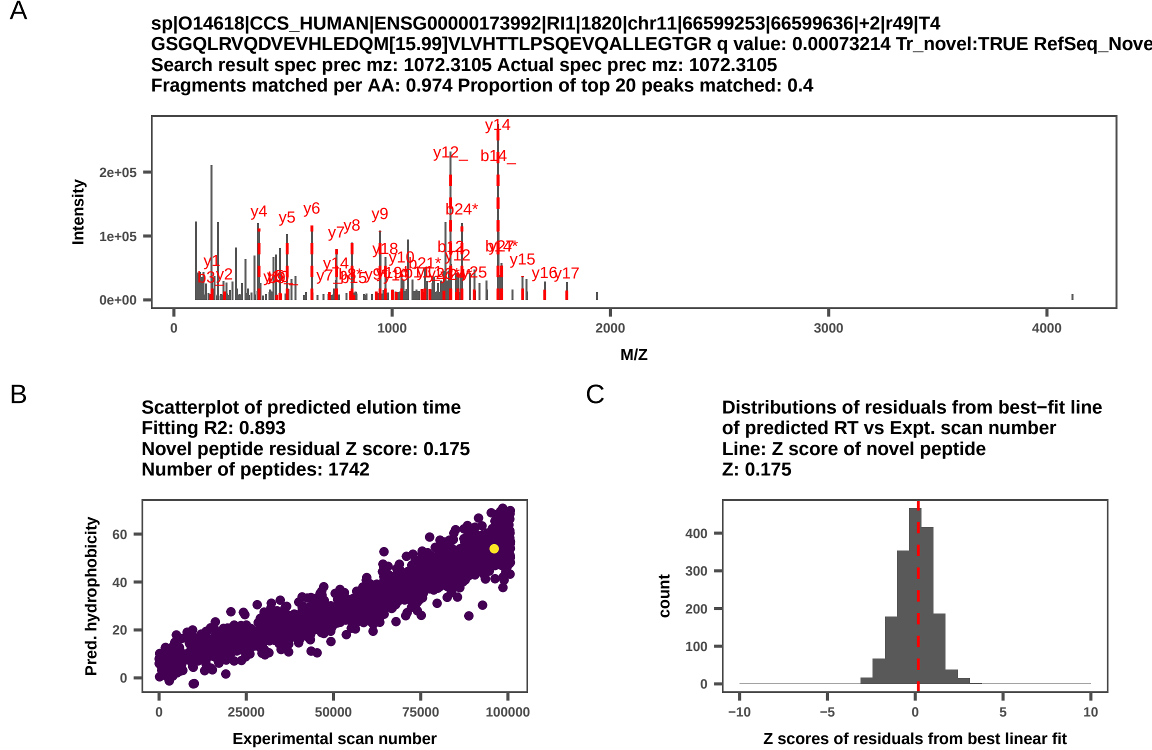
A
sp|O14618|CCS_HUMAN|ENSG00000173992|RI1|1820|chr11|66599253|66599636|+2|r49|T4
GSGQLRVQDVEVHLEDQM[15.99]VLVHTTLPSQEVQALLEGTGR q value: 0.00073214 Tr_novel:TRUE RefSeq_Nove
Search result spec prec mz: 1072.3105 Actual spec prec mz: 1072.3105
Fragments matched per AA: 0.974 Proportion of top 20 peaks matched: 0.4
Intensity
2e+05
1e+05
0e+00
y14
y12_
b14_
b24*
y4
y5
y6
y7
y8
y9
y18
y10
b21*
b12
y12
y15
y16
y17
y1
y2
b3
0
1000
2000
3000
4000
M/Z
B
Scatterplot of predicted elution time
Fitting R2: 0.893
Novel peptide residual Z score: 0.175
Number of peptides: 1742
Pred. hydrophobicity
60
40
20
0
0
25000
50000
75000
100000
Experimental scan number
C
Distributions of residuals from best−fit line
of predicted RT vs Expt. scan number
Line: Z score of novel peptide
Z: 0.175
count
400
300
200
100
0
−10
−5
0
5
10
Z scores of residuals from best linear fit
